# Assessing the Recording of Adequate Care Plans in Child and Adolescent Mental Health Services: A Clinical Audit

**DOI:** 10.1192/bjo.2025.10563

**Published:** 2025-06-20

**Authors:** Sabrina Choudary, Hana Yokoyama-King, Annabel Ariyathurai, Kiruthika Sivasubramanian

**Affiliations:** 1 University of Birmingham, Birmingham, United Kingdom; 2 Sandwell CAMHS, Black Country Healthcare NHS Foundation Trust, Birmingham, United Kingdom

## Abstract

**Aims:** Care plans can be integral to community psychiatric services to evidence personalised care through shared decision-making. Black Country Healthcare NHS Foundation Trust (BCHFT) guidelines require a documented care plan for each patient, outlining their needs, goals and preferences. Additionally, the GMC advises doctors to keep contemporaneous records for children and young people. This maintains clarity with the patient, their family, GP, and the wider multidisciplinary team.

This audit aimed to evaluate whether doctors’ care plans at Sandwell CAMHS aligned with BCHFT guidelines, providing insight into their quality and completeness.

**Methods:** From the doctors’ caseloads, 40 patients aged 18 and under were selected using a randomised generator. The data was collected retrospectively by reviewing the most recent outpatient clinic letters on electronic patient records from the past 12 months. The focus was identifying whether 5 key criteria from the local guidelines were covered in the care plans: ‘My Medication and Treatment’ (including psychological therapies); ‘My Education/training’; ‘My Physical Health’; and ‘When I Need Urgent Support’.

**Results:** The majority of care plans included medication information (79%) when relevant, demonstrating good adherence to local guidelines. However, only few care plans included documentation of physical health considerations (36%) and crisis planning (20%). 15% of letters did not have a clear ‘Care Plan’ subheading.

**Conclusion:** Care plans at Sandwell CAHMS do not currently fully comply with local guidelines across 5 criteria. Although care plans are by nature individualised, and hence subjective, we suggest implementing a standardised template for clinic letters that doctors could adjust according to the patient context. A specific subtitled section ‘Care Plan’ would help to make information clearer for the patient and other healthcare professionals. Local crisis contacts and safety netting information could be included as standard on every clinic letter. Re-audit following implementation of these recommendations will complete the audit cycle.

